# Antioxidant and Anti-Inflammatory Effects of Oral Supplementation with a Highly-Concentrated Docosahexaenoic Acid (DHA) Triglyceride in Patients with Keratoconus: A Randomized Controlled Preliminary Study

**DOI:** 10.3390/nu15051300

**Published:** 2023-03-06

**Authors:** Cristina Peris-Martínez, José Vicente Piá-Ludeña, María José Rog-Revert, Ester Fernández-López, Joan Carles Domingo

**Affiliations:** 1 Unit of Cornea and Anterior Eye Diseases, FISABIO Medical Ophthalmology (FOM), C/Pío Baroja 12, E-46015 Valencia, Spain; 2 Department of Surgery, Ophthalmology, Universitat de Valencia, Avenida Blasco Ibáñez 15, E-46010 Valencia, Spain; 3 Aviñó Peris Eye Clinic, Avenida del Oeste 34, E-46001 Valencia, Spain; 4 Department of Biochemistry and Molecular Biomedicine, Faculty of Biology, University of Barcelona, Avinguda Diagonal 643, E-08028 Barcelona, Spain

**Keywords:** keratoconus, docosahexaenoic acid, oxidative stress, omega-3 fatty acids, anti-inflammatory

## Abstract

A prospective, randomized, single-center preliminary study was performed in patients with keratoconus stages I–III (Amsler–Krumeich), who received a high rich docosahexaenoic acid (DHA) (1000 mg/day) supplement for 3 months versus untreated patients. One eye per patient was evaluated. Thirty-four patients were recruited (75% men, mean age 31 years), with 15 randomized to the control group and 19 to the DHA-treated group. Corneal topography variables and plasma biomarkers of oxidative stress and inflammatory status were evaluated. A panel of fatty acids in blood samples was also assessed. There were significant between-group differences in the astigmatism axis, asphericity coefficient, and intraocular pressure in favor of the DHA group. Additionally, between-group significant differences in total antioxidant capacity (TAC), malondialdehyde (MDA), free glutathione (GSH) and GSH/GSSG ratio, as well as reduced values of inflammatory markers, including interleukin (IL)-4, IL-6, and vascular endothelial growth factor (VEGF-A) were found. These preliminary findings support the usefulness of the antioxidant and anti-inflammatory effects of DHA supplementation for targeting underlying pathophysiological mechanisms of keratoconus. Prolonged duration of DHA supplementation may be needed to detect more noticeable clinical changes in corneal topography.

## 1. Introduction

Keratoconus is a multifactorial ectatic corneal disorder, characterized by a progressive process of corneal thinning and steeping leading to irregular astigmatism with decreased visual acuity. Keratoconus is a complex condition, and a wide variety of both genetic and environmental factors have been identified in the etiology of the disease [1,2]; however, the specific pathophysiological mechanisms remain ambiguous [3]. Traditionally, the condition has been described as a non-inflammatory disease since keratoconic corneas are strikingly lacking histological and clinical features of inflammation, such as cellular infiltration and neovascularization [4,5]. Recent studies, however, have shown an alteration in the expression of molecules involved in inflammatory processes [5,6], oxidative stress [7,8], extracellular matrix proteolysis, degradation of the corneal collagen, disturbed regulation of the corneal microenvironment [9], and cellular apoptosis [10,11], evidencing the participation of all these biological mechanisms in the pathogenesis of keratoconus. In addition, lipid mediators along with fatty acids (such as stearic, oleic, and palmitic acids) are one of the main components of human cornea and are involved in complex processes associated with inflammatory reactions in corneal injury and repair [12]. The profiling of the metabolome of keratoconus has also revealed a metabolomics signature that discriminates keratoconus from the normal cornea [13].

Regardless of different treatment modalities of corneal surgery, particularly for advanced corneal ectasias [14], the involvement of inflammatory mediators (interleukins (IL) and tumor necrosis factor alpha (TNF-α)), matrix metalloproteinases (MMP-9), oxidative stress-related products, and nutritional and/or metabolic imbalance, affecting a variety of metabolites, hormones, micronutrients, vitamins, minerals, and fatty acids [3] has been the rationale of including diet changes and nutritional supplementation in traditional conservative management of keratoconus [15].

A systematic review and meta-analysis showed that patients with keratoconus, as compared with controls, had significantly lower levels of vitamin D, zinc, and selenium levels [16]. In a prospective observational pilot study of 20 patients with keratoconus and vitamin D deficiency, vitamin D supplementation increased cell availability of copper and stabilized the disease in nearly two-thirds of the eyes [17]. In another study, decreased vitamin D levels significantly increased non-progressive keratoconus probability by 1.23 times and progressive keratoconus probability by 1.29 times more than the control group [18]. On the other hand, reduced levels of vitamin D, copper, zinc, and selenium have been shown in a comparative study of patients with keratoconus and age-matched healthy subjects [19]. Arginine supplementation in a model of human corneal fibroblasts improved extracellular matrix secretion and deposition by keratoconus cells [20]. Keratoconus dietary supplements based on antioxidant properties of vitamins and minerals are available in the market as over the counter popular corneal protection formulas.

Among omega-3 polyunsaturated fatty acids (PUFAs), docosahexaenoic acid (DHA, C22:6 *n*-3), a critical component of cell membrane phospholipids, exerts pleiotropic effects at both central and peripheral levels with health benefits in many aspects of neuronal, immune, cognitive, and cardiovascular functions [21,22,23]. Clinical studies of dietary supplementation with a highly concentrated DHA triglyceride have shown consistent anti-inflammatory, antioxidant, antiangiogenic, and antiproliferative effects targeting pathophysiological pathways involved in different eye diseases [24], including diabetic retinopathy and macular edema [22,23,24,25,26,27,28], ocular surface disorders [29,30], meibomian gland dysfunction [31,32], and pseudoexfoliative glaucoma [33].

Based on this experience, it was considered of interest to explore the antioxidant and anti-inflammatory potential of a highly concentrated DHA triglyceride product in patients with keratoconus. For this purpose, a prospective preliminary study was designed to assess the effects of 3-month DHA nutritional supplementation on clinical variables, and inflammatory and oxidative stress biomarkers of patients with early and moderate keratoconus.

## 2. Materials and Methods

### 2.1. Design and Participants

This was a single-center, prospective, randomized, and controlled preliminary study carried out between February 2019 and January 2022 at the Unit of Corneal and Anterior Eye Diseases of FISABIO Medical Ophthalmology Center (FOM) in Valencia, Spain. The primary objective of the study was to determine the effect of daily supplementation with a nutraceutical formulation of a highly concentrated DHA triglyceride plus minerals on ophthalmological parameters and biomarkers of oxidative stress and inflammation in blood samples. Secondary objectives were to assess changes in lipidomic biomarkers and correlations between ophthalmological variables and biomarkers of oxidative stress and inflammation in patients treated with the nutraceutical product.

Eligible patients were men or women aged 18 years or older, diagnosed with keratoconus stages I to III according to the Amsler–Krumeich classification [34], non-contact lens wearers, without history of previous corneal surgery, capacity to volunteer, and willing and able to follow the study protocol. The diagnosis of keratoconus was made by one experienced clinician (C.P.-M.) based on typical ophthalmological features on corneal topography and at least one keratoconus sign on slit-lamp examination [35].

Exclusion criteria were as follows: advanced keratoconus (stage IV of the Amsler–Krumeich classification); presence of other ectasias (including iatrogenic ectasia secondary to ocular surface surgery with excimer laser, radial keratotomy, traumatic corneal ectasia, etc.); eyelid alterations; previous ocular surgery; any ocular or systemic condition that may affect the interpretation of results; glaucoma or ocular hypertension; history of ocular trauma, infection, or inflammation; current treatment with topical or systemic anti-inflammatory drugs; use of nutritional supplements including omega-3 fatty acids, vitamins, and minerals (unless a washout period of 1 month has been established); hypersensitivity to fish proteins; pregnant women; refusal to sign the written informed consent; and patients deemed ineligible by the ophthalmologist.

The study was conducted in accordance with the Declaration of Helsinki and approved by the Clinical Research Ethics Committee (CEIC) of FISABIO Medical Ophthalmology Center and the Foundation for the Promotion of Health and Biomedical Research in the Community of Valencia (protocol code PI_77, approval date 26 July 2018) (Valencia, Spain). All participants signed the written informed consent form.

### 2.2. Intervention

Patients who met the inclusion criteria were assigned a consecutive number according to the order of arrival, and then randomized to treatment with the nutraceutical DHA supplement (DHA group; even number) or no supplementation (control group; odd number). Patients randomized to the DHA group received a high dose DHA formulation (Tridocosahexanoin-AOX^®^ 70%) (Brudyitis^®^, Brudy Lab, S.L., Barcelona, Spain). This is a highly concentrated DHA triglyceride having a high antioxidant activity patented to prevent cellular oxidative damage [36,37]. The composition of the product includes 602 mg of omega-3 PUFAs, 500 mg of which are DHA triglyceride, 61 mg eicosapentaenoic acid (EPA), and 42 mg docosapentaenoic acid (DPA), a mixture of essential trace elements (zinc 5 mg, selenium 27.5 µg, copper 0.5 mg, and manganese 1 mg), and glutathione 5 mg. This supplement is registered as a food supplement in the Spanish Agency for Food Safety and Nutrition (AESAN). Patients were advised to take two capsules of the supplement once daily preferably at the time of breakfast. The duration of treatment was 3 months.

### 2.3. Study Procedures

The study included a selection visit 14 days before entering the study to check the inclusion criteria, provide full information of the characteristics of the study, and perform a standard medical history. The selection visit was followed by a baseline visit (visit 0), a visit on day 14 (±3 days) (visit 1), and a final visit on day 90 (±7 days) (visit 2). At baseline, the following ophthalmological examinations were performed: corrected and uncorrected visual acuity using an ETDRS optotype at 2 m distance from the observer; slit lamp biomicroscopy; corneal topography (Pentacam^®^ HR, Oculus Inc., Arlington, WA, USA); and measurement of intraocular pressure (IOP) using a pneumotonometer (Goldmann applanation tonometer) and rebound tonometer (iCare^®^). In addition, a blood sample from a peripheral vein in fasting conditions was drawn for laboratory tests. In patients assigned to the DHA group, three boxes of the nutraceutical product were provided for 45 days of treatment. At visit 1, baseline ophthalmological examinations were repeated, and patients assigned to the DHA group received the corresponding supplement for treatment during the remaining 45 days until the end of the study. At visit 2 (end of study), the same ophthalmological examination was performed, and blood samples were collected. Capsules returned at visit 1 and at the final visit were counted to determine adherence to the study treatment. Adherence to treatment was considered when at least 80% of capsules had been consumed. Tolerance and product safety were assessed at the end of the study.

Ophthalmological variables were flat keratometry (K1, measured in diopters); steep keratometry (K2, diopters); maximum keratometry (Kmax, diopters); mean keratometry (Km, diopters); astigmatism axis (in degrees); degree of astigmatism (diopters); asphericity coefficient (Q) in the horizontal and vertical hemi-axes; corneal apex thickness (CAT, µm); central corneal thickness (CCT, µm); minimum corneal thickness (MCT, µm); chromatic aberration (CA, mm); IOP Goldmann applanation tonometer (GAT, mmHg); and IOP rebound (RBT, mmHg).

Biochemical variables included lipidomic biomarkers, antioxidant biomarkers, and inflammatory biomarkers. The panel of fatty acids included the components of the main families of saturated fatty acids (SFAs) (myristic acid, palmitic acid, stearic acid, arachidic acid, behenic acid, and lignoceric acid); monounsaturated fatty acids (MUFAs) (palmitoleic acid, oleic acid, cis-vaccenic acid, gondoic acid, erucic acid, and nervonic acid); *n*-6 PUFAs (linoleic acid, γ-linoleic acid, eicosadienoic acid, dihomo-γ-linolenic acid, arachidonic acid (ARA), adrenic acid, and osbond acid or docosapentaenoic acid); and *n*-3 PUFAs (α-linoleic acid, eicosapentaenoic acid (EPA), docosapentaenoic acid, and DHA). In addition, other fatty acids ratios were calculated as *n*-6 PUFA/*n*-3 PUFA and omega-3 index (EPA + DHA).

Antioxidant activity biomarkers were total antioxidant capacity (TAC), malondialdehyde (MDA), total glutathione (GSH), free GSH, and GSH/glutathione disulfide (GSSG).

Biomarkers of inflammation included IL-1β, IL-4, IL-6, IL-10, TNF-α, and vascular endothelial growth factor-A (VEGF-A).

### 2.4. Laboratory Analyses

Gas chromatography (GC) was used for the analysis of fatty acids. Technical details included a gas chromatograph mass spectrometer (GCMS-QP2010Plus) and auto injector and autosampler (all from Shimadzu, Tokyo, Japan); a high polarity capillary column (internal diameter 15 m × 0.10 mm, film thickness 0.10 µm) (Suprawax-280, Teknokroma Analítica, S.A., Barcelona, Spain); and GCMS solution software for data acquisition. In order to optimize the whole spectrum of fatty acid analysis, functioning conditions of MS operating parameters were optimized (10,000 amu/s for scan rate, 40–400 *m*/*z* for mass range, 1.0 kV for capillary voltage). Temperatures were set at 255 and 200 °C for the interface and ion source, respectively. The peaks of fatty acid methyl esters (FAMEs) were identified through electron ionization mass spectra using NIST11 library and through GC retention times, compared with a reference FAME mixture (GLC-744, Nu-Che Prep. Inc., Elysian, MN, USA). The results were expressed in relative amounts (percentage molar of total fatty acids) of duplicate sampling.

Total antioxidant capacity (TAC) expressed as µM copper-reducing equivalents (Cat. No. STA-360) and lipid peroxidation (thiobarbituric acid reactive substances (TBARS)) assessed as malondialdehyde (MDA) levels (Cat. No. STA-330) were measured in plasma samples using the OxiSelect™ assay kit (Cell Biolabs, San Diego, CA, USA) following the manufacturer’s instructions.

The OxiSelect™ assay (Cell Biolabs, San Diego, CA, USA) was used to measure total antioxidant capacity (TAC) and malondialdehyde (MDA) levels in plasma samples. MDA levels were indicative of lipid peroxidation (thiobarbituric acid reactive substances (TBARS)). The manufacturer’s instructions were followed. TAC levels were expressed as µM copper-reducing equivalents and MDA as µM.

A fluorescently labeled microsphere-based multiplex immunoassay was used for simultaneous analysis of IL-1β, IL-4, IL-6, IL-10, IL-18, TNF-α, and VEGF-A in plasma samples. Fluorescence was read on the Luminex-100 ISv2 system (Cat. No. HCYTOMAG-60K-05, Milliplex Map Human Cytokine/Chemokine; Linco Research/Millipore, Saint-Charles, MO, USA). The intra- and inter-assay coefficient of variation for each cytokine was: IL-1β: 7 and 12%; IL-4: 3 and 11%; IL-6: 2 and 10%; IL-10: 2 and 11%; IL-18: 2 and 11%; TNF-α: 3 and 19%; and VEGF-A: 3 and 15%, respectively.

To determine glutathione levels, the DetectX^®^ Glutathione kit (Cat. No. K006) (Arbor Assays, MI, USA) validated for red blood cells or erythrocytes, was used, following the manufacturer’s instructions. The kit is designed to measure quantitatively free or reduced glutathione (GSH) and oxidized glutathione (GSSG). Total glutathione is the sum of GSH and GSSG. The measurement of glutathione is carried out by reading the fluorescence after the reaction of the reagents present in the kit with the different samples, at an emission wavelength of 510 nm and an excitation wavelength of 390 nm. Results are calculated using the means of the readings for each sample, control, and standard. The calibration curve is generated by data reduction with fit to a 4-parameter logistic curve (4PLC). The concentration values obtained are multiplied by the dilution factor used and finally normalized by dividing them by protein values obtained in the BCA protein assay.

### 2.5. Statistical Analysis

In the analysis, one eye per patient was included. In patients with bilateral keratoconus, the eye with the most advanced stage was selected. Data of patients who completed the 3-month study period were analyzed. Categorical variables are expressed as frequencies and percentages, and quantitative variables as mean and standard deviation (SD) or standard error of the mean (SEM). For the comparison of categorical variables, the chi-square test or the Fisher’s exact test was used, and for the comparison of continuous variables, the Student’s *t* test was applied. In both study groups (DHA and controls), within-group mean differences between variables at baseline and at the end of the study were compared with the Student’s *t* test for paired samples. The Student’s *t* test for independent samples (Welch’s *t* test) was used for the analysis of between-group differences at baseline and at the final visit (end of study). Statistical significance was set at *p* < 0.05. Correlations between ophthalmological variables and oxidative stress and inflammatory biomarkers in DHA-treated patients were analyzed with the Spearman’s correlation coefficient. The R (R Core Team, 2022) statistical package was used for the analysis of ophthalmological variables, and the Statistical Package for the Social Sciences (SPSS) version 25.0 (IBM Corp., Armonk, NY, USA) was used for the analysis of biochemical variables.

## 3. Results

### 3.1. Baseline Data of Patients

During the study period, 34 patients were diagnosed of keratoconus, met the inclusion criteria, and attended all study visits. There were 25 men and 9 women, with a mean (SD) age of 31 (10) years. The mean time elapsed since the diagnosis of keratoconus was 3.7 (2.8) months. At the beginning of the study, 19 patients were allocated to the DHA group and 19 to the control group. However, four patients with unilateral keratoconus did not complete visits 1 and/or 2 and were excluded from the analysis. The final study population included 19 patients in the DHA group and 15 in the control group (Table 1). The distribution of baseline variables was similar in patients assigned to the DHA group and in those assigned to the control group.

### 3.2. Changes of Ophthalmological Variables

Results of ophthalmological variables at baseline and at the end of the study are shown in Table 2. In the control group, statistically significant differences in the within-group comparisons were not found, except for a significant increase in CCT (*p* = 0.045). In the DHA group, within-group differences were not found in any ophthalmological measures, although IOP (GAT) showed a decrease, which was marginally significant (*p* = 0.052).

In the analysis of between-group differences, values of the astigmatism axis were significantly lower in the DHA group both at baseline (*p* = 0.006) and at the end of the study (*p* = 0.021) as compared with the control group. Additionally, D values of the asphericity coefficient both in the horizontal and vertical hemi-axes were significantly lower in the DHA group (Table 2).

### 3.3. Changes of Biochemical Parameters

#### 3.3.1. Lipidomic Variables

In the analysis of the panel of fatty acids, values at baseline and at the end of the study showed negligible changes either in the DHA group or in the control group for individual fatty acids of the SFA and MUFA families (data not shown). Among *n*-6 PUFAs and *n*-3 PUFAs, noticeable changes were only observed in ARA and DHA values. As shown in Table 3, the supplementation with DHA was associated with statistically significant within-group and between-group differences as compared with the control group. In controls, however, ARA levels showed a significant decrease at the end of the study as compared with baseline.

#### 3.3.2. Antioxidant Variables

Results of antioxidant variables are shown in Table 4. Plasma TAC levels showed a statistically significant increase in the DHA group with within-group and between-group differences, whereas MDA levels decreased significantly, with within-group and between-group differences. The GSH/GSSG ratio decreased in both study groups with statistically significant differences in the within-group comparisons. Values of GSH/GSSG ratio at the end of the study were significantly higher in the DHA group as compared with the control group.

**Figure 1:**
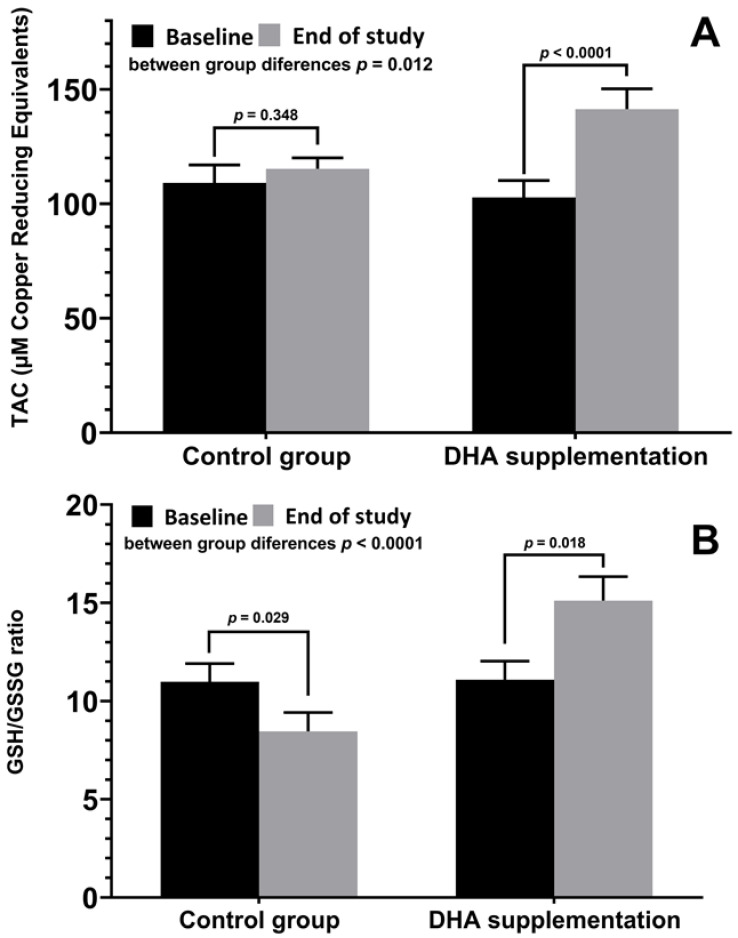
(**A**) Changes of plasma levels of TAC (mean ± SD) at baseline and at the end of the study in patients treated with DHA supplementation and controls, with significant increases in the DHA group (*p* < 0.0001) and significant between-group differences (*p* = 0.012). (**B**) Changes of GSH/GSSG ratio (mean ± SD), with a significant decrease in controls (*p* = 0.029) as opposed to a significant increase in the DHA group (*p* = 0.018), with significant between-group differences (*p* < 0.0001).

Changes in plasma TAC levels and GSH/GSSG ratio in the two study groups are shown in Figure 1.

#### 3.3.3. Inflammation-Related Variables

**Figure 2:**
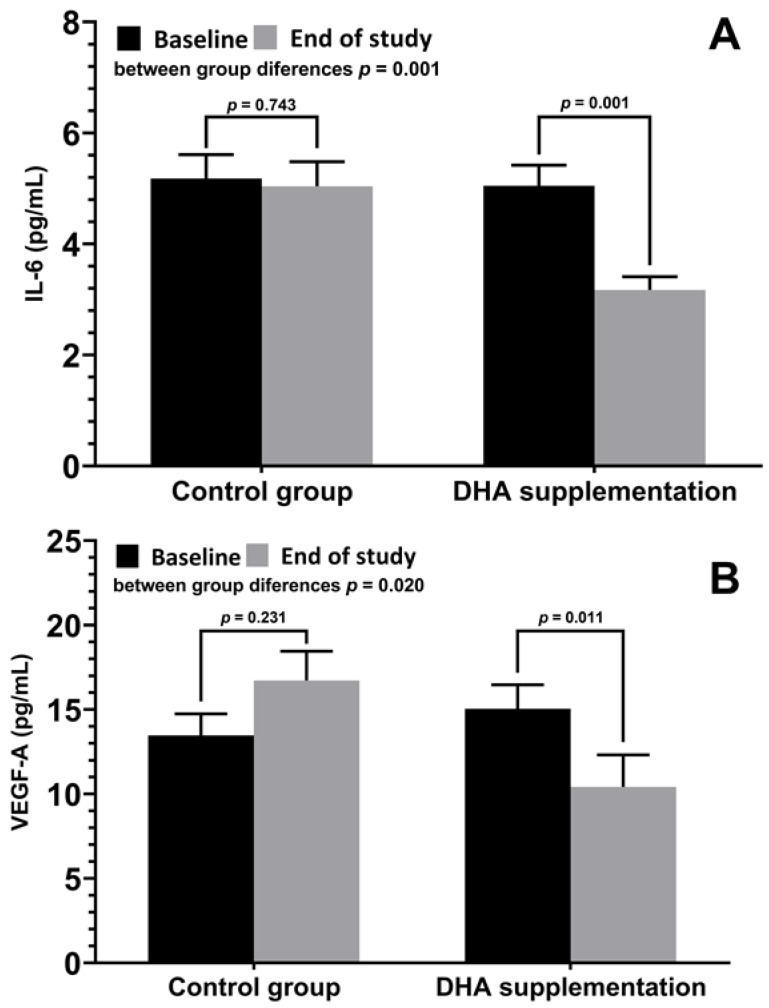
(**A**) Changes of plasma levels of IL-6 (mean ± SD) at baseline and at the end of the study in patients treated with DHA supplementation and controls, with significant decreases in the DHA group (*p* = 0.001) and significant between-group differences (*p* = 0.001). (**B**) Changes of VEGF-A (mean ± SD), with a significant decrease in the DHA group (*p* = 0.011) and with significant between-group differences (*p* = 0.020).

Results of variables related to the inflammatory status are shown in Table 5. In the group of patients treated with the DHA supplement, there were statistically significant decreases in IL-6, TNF-α, and VEGF-A at the final visit as compared with baseline, whereas in the control group, there were significant increases in IL-1β, IL-4, and IL-10 at the end of the study as compared with baseline. Statistically significant differences in the between-group comparisons were found for IL-4, IL-6, and VEGF-A with lower values in the DHA group (Figure 2).

### 3.4. Correlations between Ophthalmological Variables and Biomarkers of Oxidative Stress and Inflammation

Table 6 shows significant direct and inverse correlations between ophthalmological variables and biomarkers of oxidative stress and inflammation in patients assigned to the DHA supplementation group. The strongest positive correlations were found between K2 and IL-4 levels, and between IOP (GAT) and GSH and GSH/GSSG ratio. Negative correlation included K1 with GSH/GSSG ratio, astigmatism axis with TNF-α, and CCT with IL-6.

Finally, adherence to the active study product was greater than 80%, and adverse events were not registered in any of the patients independently of the group to which they were assigned.

## 4. Discussion

This prospective randomized study conducted in patients with keratoconus stages I-III of the Amsler–Krumeich classification was designed to assess whether the antioxidant and anti-inflammatory effects of the omega-3 fatty acid, DHA, may result in an amelioration of some ophthalmological parameters recorded by corneal topography as compared to patients who did not receive the nutraceutical supplementation. The duration of the study was 3 months. The effect of DHA on concentrations of biomarkers of oxidative stress and inflammation in blood samples was also evaluated.

Changes in keratometry parameters were not observed in any of the study groups when readings at the end of the study were compared with baseline. The astigmatism axis did not show within-group differences, but there were between-group differences at baseline and at the end of the study, with higher values in the control group. Similar findings in relation to the asphericity coefficient were found. In subjects assigned to the control group, there was a significant increase in CCT at the end of the study, whereas an increase in CCT was not observed among DHA-treated patients. The IOP measured by Goldmann applanation tonometry showed a decrease in the DHA group, which almost reached statistical significance (*p* = 0.052), but between-group differences were not observed.

In line with the antioxidative and anti-inflammatory effects of DHA, we found significant differences in the comparison between the study groups regarding plasma levels of TAC, MDA, free GSH, and GSH/GSSG ratio, as well as reduced values of inflammatory markers, including IL-4, IL-6, and VEGF-A. These observations are consistent with data obtained in previous studies carried out in different eye diseases (such as non-proliferative diabetic retinopathy, diabetic macular edema, dry eye, meibomian gland dysfunction, or exfoliative glaucoma) with the use of this highly concentrated DHA triglyceride as a nutraceutical supplement [24,25,26,27,28,29,30,31,32,33].

Overexpression of tear inflammatory cytokines in patients with keratoconus has been reported in different studies [38,39,40,41]. In a systematic review and meta-analysis of case-control and cross-sectional studies with 374 patients (374 eyes) with keratoconus showed tear levels of IL-1β, IL-6, and TNF-α significantly increased in keratoconus compared with normal controls, with standardized mean differences of 1.93 (95% CI 0.22 to 3.65, *p* = 0.03) for IL-1β, 1.22 (95% CI 0.59 to 1.84, *p* < 0.001) for IL-6, and 1.75 (95% CI 0.66 to 2.83, *p* = 0.002) for TNF-α [42]. Moreover, overexpression of IL-6 and TNF-α in tears of subclinical keratoconus [43] indicate that chronic inflammatory events are involved in the pathogenesis of keratoconus. It should be noted that in the present study, analysis of inflammatory-related markers in tear samples was planned. However, problems related to adequate amount of tear fluid sampling prevented a complete analysis of inflammatory biomarkers both in control and patients supplemented with DHA. Failure to collect adequate tear samples was partly due to logistic reasons, particularly lockdown restrictions and limited visiting access to the hospital during the COVID-19 pandemic.

In addition to the impossibility of assessing concentrations of inflammatory markers in tear samples, limitations of the study include the single-center design, the small study population, and the short duration of supplementation (only 3 months). Recruitment of eligible patients was also difficult due to the impact of the COVID-19 pandemic on healthcare. Novelty of the study, however, relies on the fact that supplementation with a highly concentrated DHA product in patients with keratoconus has not been previously evaluated. Topical omega-3 PUFA proved to be beneficial in association with a faster regeneration of corneal nerve fibers in patients with keratoconus after epithelium-off corneal collagen cross-linking [44]. In a recent study, topical omega-3 increased tear film stability more prominently than sodium hyaluronate following cross-linking [45].

Although a significant effect of DHA supplementation in the overall amelioration of corneal topography parameters was not observed, it may be argued that the antioxidant and especially the anti-inflammatory effects of DHA may not be sufficiently selective for clinical detection when targeting specific underlying mechanisms involved in early-moderate stages of keratoconus. On the other hand, it is also possible that DHA supplementation for only 3 months may be a short time to elicit clinically apparent changes. Although the mean age of the patients was 31 years, it is important to note that the patients with keratoconus aged about 20 are in a quick progress process and should be instructed about the risk of progression associated with age.

Based on the present preliminary results, a multicenter study extended for more than 1 year would be helpful to assess the long-term effect of DHA supplementation in patients with keratoconus. An international follow-up study would be particularly desirable as keratoconus parameters differ in different ethnicities [46] and by certain demographics (e.g., Down’s syndrome) [47].

## 5. Conclusions

In patients with mild to moderate keratoconus, daily supplementation with a highly-concentrated DHA triglyceride (1000 mg/day) nutritional supplement for 3 months was associated with significant improvements in antioxidant (TAC, MDA, GSH/GSSG) and inflammatory status (IL-4, IL-6, TNF-α, VEGF-A) biomarkers as compared with untreated controls. Prolonged duration of DHA supplementation may be needed to detect more noticeable clinical changes of corneal topography-related measures.

## Figures and Tables

**Table 1:** Baseline demographic and clinical data of 34 patients with keratoconus.

Variables	All Patients (*n* = 34)	Control Group (*n* = 15)	DHA Group (*n* = 19)	*p* Value
Gender, *n* (%)				0.638
Men	25 (73.5)	12 (80)	13 (68.4)	
Women	9 (26.5)	3 (20)	6 (31.6)	
Age, years, mean (SD)	31 (10)	33 (9)	30 (11)	0.484
Time since diagnosis, months, mean (SD)	3.7 (2.8)	4.3 (4.1)	3.3 (0.8)	0.366

**Table 2:** Ophthalmological variables at baseline and at the end of the study in patients treated with the DHA supplement and in controls.

Variables	Control Group (*n* = 15)		DHA Group (*n* = 19)		Within-Group *p* Value		Between-Group *p* Value	
	Baseline	End of Study	Baseline	End of Study	Control Group	DHA Group	Baseline	End of Study
K1, diopters	43.5 (3.0)	43.6 (3.1)	44.9 (2.9)	44.0 (2.8)	0.571	0.387	0.176	0.163
K2, diopters	47.0 (3.5)	47.1 (3.4)	48.8 (3.8)	48.8 (3.7)	0.221	0.779	0.155	0.174
Kmax, diopters	53.2 (6.0)	53.2 (5.8)	54.9 (5.6)	54.8 (5.6)	0.671	0.271	0.407	0.440
Km, diopters	45.1 (3.1)	45,2 (3.1)	46.8 (3.0)	46.8 (2.9)	0.299	0.409	0.147	0.145
Astigmatism axis, degrees	124.4 (52.7)	119.7 (58.4)	66.0 (63.6)	69.7 (60.8)	0.369	0.310	**0.006**	**0.021**
Astigmatism degree diopters	3.4 (1.6)	3.5 (1.7)	3.8 (2.7)	3.8 (2.7)	0.539	0.494	0.578	0.711
Asphericity coefficient (D)								
Horizontal hemi-axis	−0.4 (0.4)	−0.4 (0.4)	−0.7 (0.3)	−0.7 (0.3)	0.612	0.119	**0.022**	**0.015**
Vertical hemi-axis	−0.4 (0.3)	−0.4 (0.4)	−0.8 (0.3)	−0.8 (0.3)	0.346	0.771	**0.008**	**0.007**
CAT, µm	489.8 (38.7)	491.6 (39.2)	469,3 (46.7)	470.3 (46.7)	0.147	0.318	0.172	0.157
CCT, µm	495.8 (37.2)	498.2 (37.6)	478.1 (44.6)	478.4 (44.7)	**0.045**	0.734	0.217	0.171
MCT, µm	470.4 (51.9)	470.7 (50.6)	459.8 (46.5)	461.0 (46.4)	0.830	0.252	0.541	0.568
CA, mm	2.4 (1.8)	2.4 (1.8)	2.2 (1.3)	2.3 (1.3)	0.111	0.528	0.675	0.900
IOP GAT, mmHg	10.8 (2.4)	11.9 (4.0)	11.5 (3.2)	10.3 (3.0)	0.214	0.052	0.373	0.192
IOP rebound, mmHg	10.4 (3.3)	10.8 (2.8)	10.6 (3.2)	9.9 (3.0)	0.672	0.371	0.839	0.381

K1: flat keratometry; K2: steep keratometry; Kmax: maximum keratometry; Km: mean keratometry; CAT: corneal apex thickness; CCT: central corneal thickness; MCT: minimum corneal thickness; CA: chromatic aberration; IOP: intraocular pressure; GAT: Goldmann applanation tonometry. Data expressed as mean and standard deviation (SD).

**Table 3:** Lipidomic variables at baseline and at the end of the study in patients treated with the DHA supplement and in controls.

Variables	Control Group (*n* = 15)		DHA Group (*n* = 19)		Within-Group *p* Value		Between-Group *p* Value	
	Baseline	End of Study	Baseline	End of Study	Control Group	DHA Group	Baseline	End of Study
DHA, % total fatty acids	2.98 (0.19)	2.97 (0.18)	3.02 (0.23)	4.95 (0.13)	0.945	**<0.0001**	0.925	**<0.0001**
ARA, % total fatty acids	12.93 (0.16)	12.58 (0.20)	12.85 (0.23)	11.52 (0.25)	**0.009**	**<0.0001**	0.831	**0.005**
*n*-6 PUFA/*n*-3 PUFA	6.61 (0.50)	6.43 (0.49)	6.55 (0.51)	3.79 (0.14)	0.304	**<0.0001**	0.878	**<0.0001**
Omega-3 index	3.23 (0.21)	3.21 (0.19)	3.30 (0.26)	5.46 (0.16)	0.890	**<0.0001**	0.939	**<0.0001**

DHA: docosahexaenoic acid; ARA: arachidonic acid; PUFA: polyunsaturated fatty acids. Data expressed as mean and standard error of the mean (SEM).

**Table 4:** Antioxidant variables at baseline and at the end of the study in patients treated with the DHA supplement and in controls.

Variables	Control Group (*n* = 15)		DHA Group (*n* = 19)		Within-Group *p* Value		Between-Group *p* Value	
	Baseline	End of Study	Baseline	End of Study	Control Group	DHA Group	Baseline	End of Study
TAC, µ Cu reducing equiv.	109.20 (7.80)	115.30 (4.76)	102.80 (7.37)	141.40 (8.82)	0.348	**<0.0001**	0.556	**0.012**
MDA, µM	4.02 (0.23)	4.36 (0.29)	3.84 (0.17)	3.15 (0.24)	0.322	**0.004**	0.701	**0.0007**
GSH, total, mmol/mg protein	10.67 (0.55)	10.12 (0.50)	9.44 (0.55)	10.30 (0.52)	0.246	0.229	0.126	0.912
GSH, free, mmol/mg protein	8.87 (0.43)	7.93 (0.45)	7.90 (0.49)	9.01 (0.48)	0.118	0.121	0.224	0.071
GSH/GSSG ratio	10.98 (0.93)	8.46 (0.96)	11.09 (0.94)	15.11 (1.22)	**0.029**	**0.018**	0.986	**<0.0001**

TAC: total antioxidant capacity; Cu: copper; equiv: equivalents; MDA: malondialdehyde; GSH: glutathione; GSSG: oxidized glutathione. Data expressed as mean and standard error of the mean (SEM) in parenthesis.

**Table 5:** Variables related to inflammation at baseline and at the end of the study in patients treated with the DHA supplement and in controls.

Variables	Control Group (*n* = 15)		DHA Group (*n* = 19)		Within-Group *p* Value		Between-Group *p* Value	
	Baseline	End of Study	Baseline	End of Study	Control Group	DHA Group	Baseline	End of Study
IL-1β, pg/mL	5.02 (0.43)	7.43 (0.66)	5.21 (0.47)	6.24 (0.60)	**0.002**	0.129	0.701	0.146
IL-4, pg/mL	2.84 (0.29)	3.65 (0.34)	2.89 (0.28)	2.19 (0.22)	**0.002**	0.087	0.805	**0.0003**
IL-6, pg/mL	5.18 (0.43)	5.04 (0.44)	5.05 (0.37)	3.17 (0.24)	0.743	**0.0002**	0.714	**0.001**
IL-10, pg/mL	4.21 (0.37)	5.66 (0.52)	4.15 (0.32)	4.81 (0.54)	**0.015**	0.404	0.939	0.167
IL-18, pg/mL	73.0 (6.09)	74.97 (6.39)	80.52 (7.17)	79.74 (6.57)	0.745	0.999	0.522	0.652
TNF-α, pg/mL	39.45 (3.81)	43.29 (4.07)	58.53 (5.17)	49.36 (4.28)	0.274	**0.059**	**0.009**	0.437
VEGF-A, pg/mL	13.46 (1.28)	16.72 (1.73)	15.04 (1.42)	10.42 (1.90)	0.231	**0.011**	0.528	**0.020**

IL: interleukin; TNF: tumor necrosis factor; VEGF: vascular endothelial growth factor. Data expressed as mean and standard error of the mean (SEM).

**Table 6:** Correlations between ophthalmological parameters and biomarkers of oxidative stress and inflammation in patients with keratoconus treated with DHA supplementation.

Ophthalmological Variables	Oxidative Stress/ Inflammatory Biomarkers	Correlation Coefficient (95% Confidence Interval)
K1, flat keratometry	- GSH/GSSG ratio	−0.51 (−0.79 to −0.006)
	- IL-8	0.46 (−0.01 to 0.76)
K2, steep keratometry	- IL-4	0.65 (0.27 to 0.86)
	- IL-10	0.53 (0.09 to 0.80)
Astigmatism axis	- TNF-α	−0.49 (−0.78 to −0.03)
Astigmatism degree	- GSH free	0.47 (0.0 to 0.77)
Central corneal thickness (CCT)	- IL-6	−0.46 (−0.76 to 0.0)
Chromatic aberration (CA)	- IL-10	0.55 (0.11 to 0.81)
Intraocular pressure (IOP) (Goldmann applanation tonometry)	- GSH total	0.50 (00.5 to 0.78)
	- GSH free	0.62 (0.21 to 0.84)
	- GSH/GSSG	0.68 (0.31 to 0.87)
	- IL-18	−0.59 (−0.83 to −0.17)

GSH: glutathione; GSSG: oxidized glutathione; IL: interleukin; TNF: tumor necrosis factor.

## Data Availability

Study data are available from the corresponding author upon request.
